# First case report of short-chain acyl-CoA dehydrogenase deficiency in China

**DOI:** 10.1186/1687-9856-2013-S1-P181

**Published:** 2013-10-03

**Authors:** MinYan Jiang, Li Liu, MinZhi Peng, CuiLi Liang, HuiYing Sheng, YanNa Cai

**Affiliations:** 1 Department of Endocrinology and Metabolisms, Guangzhou Women and Children’s Medical Center, Guangzhou, Guangdong, China

## 

Short-chain acyl-CoA dehydrogenase deficiency (SCADD) is a rare autosomal recessive inborn error of mitochondrial fatty acid oxidation. It caused by rare mutations as well as polymorphic susceptibility variants. We describe the case of a 1-year-old male patient who had growth and mental retardation, seizures, fever since infancy. Urinary GC/MS showed elevated levels of ethylmalonic acid. Plasma acylcarnitines on MS/MS, elevations of C4-cartinitine are consistently present. The two polymorphic susceptibility variants of SCAD gene, c.625G>A and c.322G>A, was detected. As its highly variable clinical characteristics, there is no related report in China. This report broadens the phenotype and genotype of SCAD deficiency in China and underlines the difficulty of diagnosis.

